# Comparison the Structural, Physicochemical, and Prebiotic Properties of Litchi Pomace Dietary Fibers before and after Modification

**DOI:** 10.3390/foods11030248

**Published:** 2022-01-18

**Authors:** Yina Li, Yuanshan Yu, Jijun Wu, Yujuan Xu, Gengsheng Xiao, Lu Li, Haoran Liu

**Affiliations:** 1 Sericultural & Argi-Food Research Institute, Guangdong Academy of Agricultural Sciences/Key Laboratory of Functional Foods, Ministry of Agriculture and Rural Affairs/Guangdong Key Laboratory of Agricultural Products Processing, Guangzhou 510610, China; yara1ne@163.com (Y.L.); guoshuwujijun@163.com (J.W.); guoshuxuyujuan@163.com (Y.X.); guoshuxgs@163.com (G.X.); lilu045@163.com (L.L.); winstonliu111@163.com (H.L.); 2 College of Food Sciences, South China Agricultural University, Guangzhou 510642, China

**Keywords:** litchi pomace, dietary fiber, monosaccharide composition, structure, prebiotic activity

## Abstract

Litchi pomace, a by-product of litchi processing, is rich in dietary fiber. Soluble and insoluble dietary fibers were extracted from litchi pomace, and insoluble dietary fiber was modified by ultrasonic enzymatic treatment to obtain modified soluble and insoluble dietary fibers. The structural, physicochemical, and functional properties of the dietary fiber samples were evaluated and compared. It was found that all dietary fiber samples displayed typical polysaccharide absorption spectra, with arabinose being the most abundant monosaccharide component. Soluble dietary fibers from litchi pomace were morphologically fragmented and relatively smooth, with relatively high swelling capacity, whereas the insoluble dietary fibers possessed wrinkles and porous structures on the surface, as well as higher water holding capacity. Additionally, soluble dietary fiber content of litchi pomace was successfully increased by 6.32 ± 0.14% after ultrasonic enzymatic modification, and its arabinose content and apparent viscosity were also significantly increased. Further, the soluble dietary fibers exhibited superior radical scavenging ability and significantly stimulated the growth of probiotic bacterial species. Taken together, this study suggested that dietary fiber from litchi pomace could be a promising ingredient for functional foods industry.

## 1. Introduction

Litchi (*Litchi chinensis* Sonn.) is an evergreen tree of the *Sapindaceae* family, with the main growing countries being China, Vietnam, Thailand, and India [1]. In China, Huaizhi and Heiye are the main litchi cultivars used for further processing to produce value-added products such as dried litchi, litchi juice, and litchi wine [2]. However, the resulting pomace, pericarp, and seeds are frequently discarded, which have been reported to contain diverse nutrients and bioactive compounds, including polysaccharides, crude fiber, and polyphenols [3]. Therefore, increasing research has focused on studying the utilization of nutrients and bioactive compounds from litchi fruit waste for the reduction of environmental burden and the potential application in food and/or functional food industry [4,5,6].

Litchi pomace produced by the process is more often used for feed production. At present, research on the structural and functional properties of dietary fiber extracted from litchi pomace is limited compared to polysaccharides and polyphenols [7,8,9]. Dietary fiber is a broad category of indigestible food ingredients and roughly classified as water-soluble dietary fiber and water-insoluble dietary fiber according to their solubility in water. Specifically, water-soluble dietary fiber mainly includes pentosan, soluble hemicellulose, gum, and pectin, while cellulose, insoluble hemicellulose and lignin are the major compositions of IDF [10]. Multiple animal studies and human trials have demonstrated that dietary fiber possess several biological activities, such as improving intestinal absorption, increasing satiety, enhancing immune function, and promoting colon health, which are determined by the source of dietary fiber with different chemical structures and compositions [11]. Moreover, many studies have been conducted to modify the structure and composition of dietary fiber through different treatments to enhance and modify its function. The single modification method has its limitations, whereas the combined modification possesses a stronger modifying effect [12,13]. Nowadays, ultrasonic enzymatic treatment is more applied to modify dietary fiber, owing to its safety, low damage to the molecular structure of dietary fiber, and its effectiveness in increasing the water-soluble dietary fiber content [14].

To better utilize the litchi fruit waste and explore the potential application of litchi dietary fiber in food and/or functional food industry, the current study aimed to compare the differences in structural and functional properties of dietary fibers obtained from litchi pomace, so as to provide ideas and theoretical basis for the further processing of litchi pomace.

## 2. Materials and Methods

### 2.1. Materials

Litchi pomace (cv. Huaizhi) was provided by Guangzhou Shunchangyuan Green Food Co., Ltd. (Guangzhou, Guangdong Province, China). Prior to dietary fiber extraction, litchi pomace was desugared by *Saccharomyces cerevisiae* fermentation. Briefly, litchi pomace was heated in a water bath at 80 °C for 20 min, 0.02% (*w*/*w*) activated *Saccharomyces cerevisiae* was added after returning to room temperature, and the mixture was placed in a constant temperature incubator for fermentation at 30 °C for 24 h, then dried with the moisture content of 8 to 12% by weight using the hot air vacuum drying. The dried sample was crushed and passed through a 60-mesh sieve to obtain the desugared litchi pomace powder, which was kept in a desiccator for later use. The monosaccharide standards were HPLC grade, and were purchased from Shanghai Yuanye Biological Technology Co., LTD (Shanghai, China). Other chemicals used in this study were of analytical grade.

### 2.2. Preparation of Dietary Fiber from Litchi Pomace

Dietary fiber from litchi pomace was prepared as previously described by Wu et al. [15] with minor modifications. Initially, the desugared litchi pomace powder was mixed with distilled water (1:20, *w*:*v*) and hydrolyzed by 0.2% (*w*/*w*, 2000 U/g) α-amylase in a shaking bath (150 r/min) at pH 6.0, 60 °C for 1 h. Thereafter, 1.0% (*w*/*w*, 100 U/mg) neutral protease was added and incubated at pH 7.0, 50 °C for 1.5 h. The treated mixture was centrifuged at 6000× *g* for 10 min after the inactivation of enzyme in boiling water bath for 10 min. The resulting residue was washed with distilled water, 95% (*v*/*v*) ethanol, and acetone and then freeze-dried in a vacuum freezer to obtain the water-insoluble dietary fiber (IDF), whereas the supernatant was vacuum-concentrated (50 °C, 40 rpm) and precipitated with four times volumes of 95% ethanol at room temperature for 12 h. After centrifugation at 6000× *g* for 10 min, the precipitate was collected and dissolved in distilled water to remove ethanol by vacuum rotary evaporation before lyophilization to obtain the water-soluble dietary fiber (SDF). The yields of SDF and IDF obtained from litchi pomace were 7.28 ± 0.13% and 69.30 ± 0.78%, respectively.

### 2.3. Ultrasonic Enzymatic Modification of IDF from Litchi Pomace

Ultrasonic enzymatic modification was conducted as the following steps: Initially, IDF was mixed with distilled water (1:20, *w*:*v*) and sonicated at 450 W, 50 °C for 30 min using the ultrasonic bath (DL-800B, Zhixin, China). Subsequently, 50 µL/g of cellulase (700 EGU/g) was added, and the mixture was incubated for 3 h at 50 °C with constant shaking (150 rpm). After the incubation, the cellulase was inactivated, followed by the centrifugation of mixture, and the resulting supernatant and precipitation were treated by aforementioned process. The water-soluble dietary fiber sample obtained was marked as M-SDF, and the water-insoluble dietary fiber sample obtained was marked as M-IDF. The yields of M-SDF and M-IDF obtained from litchi pomace were 6.32 ± 0.14% and 55.67 ± 0.53%, respectively.

### 2.4. Monosaccharide Composition

The monosaccharide composition of dietary fibers from litchi pomace was measured by 1-phenyl-3-methyl-5-pyrazolone (PMP) derivatization and high-performance liquid chromatography (HPLC, Agilent 1200 series, Mundelein, IL, USA) with a Wondasil C18 column (4.6 mm × 250 mm i.d., 5 μm particle size) according to the method of Liu et al. [16]. The standard monosaccharides, including mannose, ribose, rhamnose, galactose acid, glucose, galactose, xylose, and arabinose, were used to analyze the monosaccharides in hydrolyzed samples. Chromatographic separation was carried out using 0.05 M sodium phosphate buffer (pH 6.85) and acetonitrile at a ratio of 82:18 (*v*:*v*) as mobile phase at the flow rate of 1 mL/min. The detection wavelength was set as 250 nm.

### 2.5. Scanning Electron Microscopy (SEM)

The dietary fiber samples were mounted on an aluminum stub with double-sided stick tape and coated with a 10 nm gold layer. An electron microscope (S-3000N, Hitachi Ltd., Tokyo, Japan) captured the scanning images at an accelerating voltage of 20.0 kV. The micrographs were taken at ×1000 magnification (scale bar 50 μm).

### 2.6. Fourier Transform Infrared Spectroscopy (FTIR)

Changes in the molecular structure of samples were obtained at room temperature using FTIR (VERTEX 70, Bruker, Karlsruhe, Germany). The dry sample was mixed with KBr powder under infrared irradiation and pressed into a tablet. Spectra were collected in the range of 4000–400 cm^−1^ with a resolution of 4 cm^−1^ [17].

### 2.7. Hydration Properties

Hydration properties, including the water holding capacity (WHC), swelling capacity (SC), and water solubility (WS), were determined by the methods described by Shen et al. [18]. The WHC, SC, and WS were reported as mean value of three determinations per sample.

### 2.8. Rheological Property

The viscosity of water-soluble dietary fiber samples was measured by an AR1500EX rheometer (TA Instruments Ltd., New Castle, DE, USA) equipped with a cone and plane geometry system (40 mm diameter, 1 mm gap). In steady shear tests, the steady flow behaviors of water-soluble dietary fiber samples with different concentrations (4, 8, and 12%, *w*/*v*) were measured at a 0.1–100 s^−1^ shear rate range and 25 °C.

### 2.9. The Radical Scavenging Activity

The antioxidant active ingredient was extracted on the basis of the previous method [19], and the radical scavenging activity was measured via ABTS assay as per the method of Ruiz-Torralba et al. [20] with slight modifications. Briefly, 20 µL of each sample extract/sample aqueous solution with different mass concentrations were individually placed in a 96-well polystyrene microplate, after which 200 µL of the working ABTS solution was added. The mixture was stored at room temperature in darkness for 10 min, and the absorbance was measured by the microplate reader at 734 nm. The percent scavenging activity was calculated as follows:

ABTS radical scavenging activity (%) = 100 × [1 − (A_1_ − A_2_)/A_0_]

where A_0_ is the absorbance of the ABTS solution with ethanol instead of sample, A_1_ is the absorbance of the ABTS solution with sample, and A_2_ is the absorbance of the sample with anhydrous methanol instead of ABTS solution

### 2.10. In Vitro Probiotic Activity

In this study, both *Lactobacillus acidophilus* and *Lactobacillus plantarum* were provided by the microbiology laboratory of Sericultural and Agri-Food Research Institute (Guangdong, China) and were used to evaluate the probiotic activity of SDF and M-SDF. The preparation of inoculants was determined in accordance with steps described by Okolie et al. [21]. First, the sugar-free MRS base medium was prepared as described by Safoura et al. [22]. Then, glucose (negative control), inulin (positive control), SDF, and M-SDF were added to sugar-free MRS base medium separately at concentrations of 2% (*w*/*v*), while sugar-free MRS basal medium served as a blank control. All MRS media were autoclaved at 121 °C for 20 min and cooled to 50 °C, followed by the inoculation of 2% (*v*/*v*) above bacterial suspensions and incubation at 37 °C for 24 h. Growth of each strain was monitored by measuring pH and the optical density (OD) of 10-fold diluted at 600 nm.

### 2.11. Statistical Analysis

All measurements were performed at least in triplicate, and the results were expressed as the mean values with their standard deviation. Statistical analyses were carried using the SPSS 22.0 software (SPSS Inc., Chicago, IL, USA). Data were analyzed using one-way ANOVA and Duncan’s test. *p* < 0.05 was considered to be statistically significant.

## 3. Results and Discussion

### 3.1. Structural Characterization

#### 3.1.1. Monosaccharide Composition Analysis

A total of eight monosaccharides were detected in all four dietary fiber samples, and the monosaccharide content varied among four different dietary fiber samples (Table 1). The highest content was arabinose (83.14 ± 0.39–233.11 ± 1.84 mg/g), which was higher than that reported in defatted coconut flour (11.84 ± 0.82 mg/g) and pear pomace dietary fiber (20.40 ± 6.30 mg/g) [23,24]. Wang et al. [25] found that glucose was the main monosaccharide in orange SDF (64.00 ± 1.73 mg/g), while the main monosaccharide of the SDFs from grapefruit and lemon was arabinose (100.72 ± 2.43 mg/g and 58.82 ± 1.84 mg/g). The abundance of arabinose is characteristic of dietary fiber from litchi pomace, which has been reported to have physiological activities such as regulating lipid metabolism, affecting intestinal microbiota and metabolism [26,27]. Further, SDF and M-SDF contained significantly higher levels of galacturonic acid, arabinose, rhamnose, and galactose, which were typical constituents of pectin [28], and thus it may be concluded that arabinose-rich pectin is the key component of soluble dietary fiber from litchi pomace. The high contents of arabinose, glucose, and xylose in IDF and M-IDF indicated that arabinoxylan and cellulose may be the main components of insoluble dietary fiber from litchi pomace [29]. In addition, M-SDF contained more galacturonic acid, glucose, and arabinose glucose than SDF, whereas the concentrations of galacturonic acid, xylose, and arabinose in M-IDF were lower than that in IDF, indicating that ultrasonic enzymatic modification may lead to the hydrolysis of cellulose in the cell wall and the release of some xylan hemicellulose, thus facilitating the conversion of insoluble dietary fiber to soluble dietary fiber [30].

#### 3.1.2. SEM Analysis

**Figure 1:**
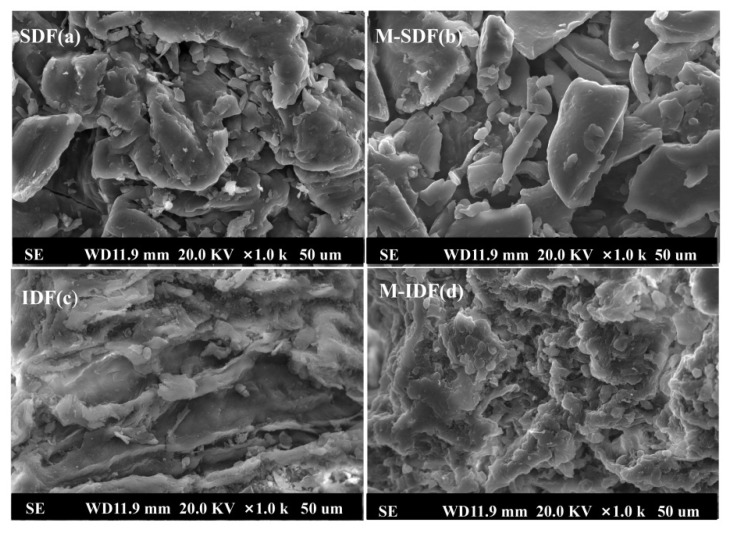
SEM images of SDF (**a**), M-SDF (**b**), IDF (**c**), and M-IDF (**d**). Magnification: 1000× (scale bar 50 μm).

As depicted in Figure 1, the morphological characterization of the dietary fiber samples at magnification times of 1000 was observed using SEM. These micro images clearly indicated the significant variations of surface topography among the dietary fiber samples. Compared to SDF, IDF had rougher topography with obvious wrinkles, which might have been the result of different dietary fibers types. In the study of Lyu et al. [31], the tissue state of high-purity insoluble fiber from soybean dregs showed hierarchical and low aggregation degree. The finding of Chen et al. [32] revealed that wheat-soluble dietary fiber had a relatively flat and loose structure with a certain gap between fibers, whereas the surface of wheat-insoluble dietary fiber was irregular with a large number of cracks and small clumps. In addition, the microstructure of the dietary fibers obtained by ultrasonic enzymatic modification showed significant changes in morphology, with M-SDF appearing more fragmented than SDF and M-IDF, presenting cellular network structure with stronger porosity than IDF, which indicated that ultrasonic enzymatic modification can destroy the matrix of cellulose, hemicellulose, and lignin, thus promoting the transformation of IDF to M-SDF [33]. Similar results were obtained by Ma et al. [17]. The physiochemical properties of dietary fiber are determined by its microstructure. Porous and folded structure can increase the specific surface area and expose more polar groups, consequently promoting the adsorption and binding of water, which further affects its application in food [34].

#### 3.1.3. FTIR Analysis

**Figure 2:**
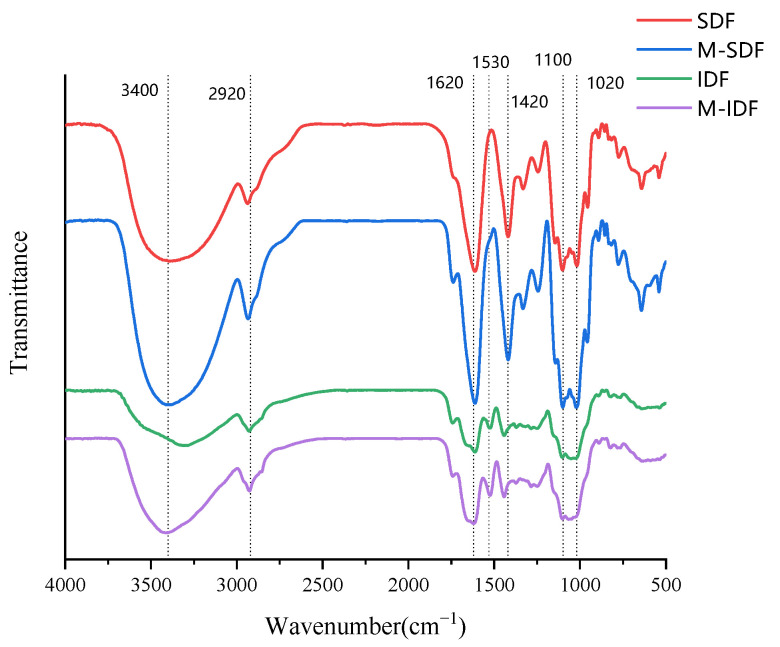
FTIR of SDF, M-SDF, IDF, and M-IDF.

The infrared spectra of all dietary fiber samples displayed the characteristic absorption peaks of cellulose polysaccharides (Figure 2). The overall peak intensity of SDF and M-SDF was significantly higher than that of IDF and M-IDF, indicating that SDF and M-SDF had more typical polysaccharide complex structures. The wide absorption band near 3400 cm^−1^ was caused by the stretching of O–H groups in polysaccharides. The peak intensity of M-SDF was relatively high at this wavelength, indicating that there were more hydrogen bonds in the associative state, which may have been related to M-SDF containing more gels (galactose acid) and hemicelluloses (mannose, glucose, galactose, and arabinose) (Table 1) [30]. The absorption peak near 1620 cm^−1^ was the stretching vibration absorption of C=O, suggesting the presence of amide groups. The absorption peak in the range of 1200–1400 cm^−1^ was caused by the angular vibrations of C–H, representing the typical structure of a carbohydrate skeleton. The absorption peak in the range of 1200–1000 cm^−1^ corresponded to the stretching vibrations of C–C, C–O, and C–O–C, which were frequently reported as the presence of sugar aldehyde groups [35]. The modified dietary fibers presented stronger absorption peaks at the wavelength of these characteristic romaegions, which indicated that the supramolecular structure and reactive groups of litchi pomace dietary fiber were changed by the ultrasonic enzymatic modification. In addition, IDF and M-IDF were observed to have characteristic absorption peaks of aromatic lignin hydrocarbons near 1530 cm^−1^ [36], while SDF and M-SDF showed clearly characteristic absorption peaks of β-glucoside bond, furanose, and α- and β- pyranose in the range of 1000–700 cm^−1^ [37].

### 3.2. Physicochemical Properties

#### 3.2.1. Hydration Properties

Hydration properties of the dietary fiber samples are presented in Table 2. WHC refers to the ability of wet materials to retain water under external force (centrifugal force or compression, etc.). SC measures the change of the total volume of materials and water, while WS reflects the degree to which materials can be dissolved by water at a certain temperature [38]. As observed from the results, SDF had the highest SC and WS, while the WHC of IDF was higher than that of SDF. These results were consistent with the findings of Zhu et al. [39], which might have been due to greater fiber porosity and hydrogen bond content in IDF. After ultrasonic enzymatic modification, the SC of M-IDF was 7.18 ± 0.25 mL/g, whereas the WHC decreased to 9.07 ± 0.09 g/g, which were higher relative to that of apple fiber (6.89 ± 0.11 mL/g and 6.12 ± 0.11 g/g) [40]. A previous work found that the SC and WHC were decreased in rice bran dietary fiber after cellulase modification [33], which was attributed to cellulase treatment changing the contents of hydrophilic components such as hemicellulose and cellulose. Hydration properties of dietary fiber are closely related to its source, structural morphology, porosity, and processing parameters [41]. Typically, the high WHC and SC indicate that litchi pomace dietary fiber may be considered for use in food processing to modify texture and avoid dehydration in formula foods.

#### 3.2.2. Rheological Properties

**Figure 3:**
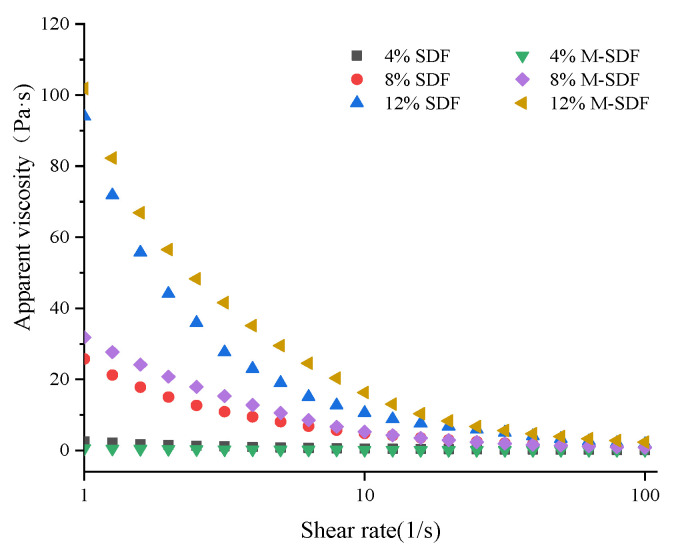
Viscosity curves of SDF and M-SDF at 4, 8, and 12% (*w*/*v*).

The steady flow test curves of SDF and M-SDF are plotted in Figure 3. The apparent viscosity of SDF and M-SDF decreased gradually with the increase of shear rate at room temperature, presenting shear dilution phenomenon, which showed that SDF and M-SDF belonged to pseudoplastic non-Newtonian fluids. Moreover, with the increase of concentration, the apparent viscosities of SDF and M-SDF increased accordingly. It can be seen that the concentration of the sample had a direct and nonlinear effect on the viscosities of the constant temperature solution, which was consistent with the results of Feng et al. [42]. This might have been due to the fact that the interaction force among the SDFs molecules elevated at high concentration, which increased the degree of cross-linking and polymerization of substances. Additionally, at the same concentration and shear rate, the apparent viscosity of M-SDF was greater than that of SDF, which may have been associated with the greater pectin structures and longer the molecular chain length of M-SDF [24,43]. Previous studies have shown that viscosity is one of the important properties of SDF, which is closely related to the ability of SDF to delay and reduce the absorption of food components in the digestive tract and reduce the postprandial blood glucose response [44]. Thus, we speculated that M-SDF may have a better in vivo physiological activity than SDF.

### 3.3. Functional Properties

#### 3.3.1. The Radical Scavenging Activity

**Figure 4:**
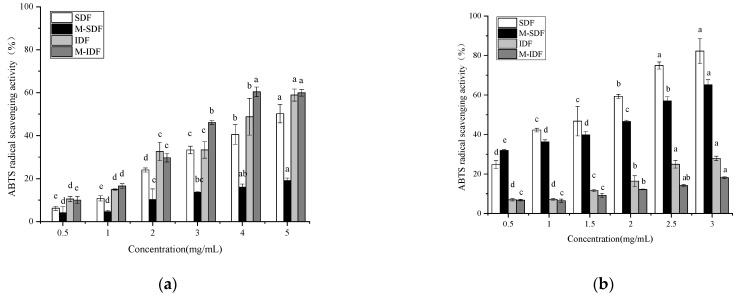
(**a**) ABTS radical scavenging activities of sample extracts; (**b**) ABTS radical scavenging activities of sample aqueous solutions. Different letters (a, b, c, d, e) in the same row indicate significantly different means at *p* < 0.05 (Duncan’s test).

As shown in Figure 4, both types of the dietary fiber samples exhibited radical scavenging activity with a clear dose-dependent manner. At the dose of 5.0 mg/mL, the ABTS radical scavenging activity of SDF, M-SDF, IDF, and M-IDF extracts were, respectively, 50.24%, 19.08%, 58.92%, and 59.95% (Figure 4a), which was similar to that reported in black mulberry [45]. Consistent with the findings of a previous work [46], IDF extract was observed to possess higher radical scavenging as compared to SDF extract, which can be attributed to a higher amount of phenols that were co-extracted with the molecule [47]. The hydroxyl groups of phenolic compounds could donate electrons or hydrogen atoms to enhance the antioxidant activity [48]. In addition, at the dose of 3.0 mg/mL, the ABTS radical scavenging activities of dietary fiber aqueous solution were 82.24%, 65.20%, 27.90%, and 18.20%, respectively (Figure 4b). The radical scavenging activity of SDF and M-SDF aqueous solution was significantly higher than that of IDF and M-IDF aqueous solution, which was probably due to its higher content of uronic acid and the presence of hydroxyl groups in the main backbone [32]. SDF possessed relatively higher radical scavenging capacity than M-SDF, which may be related to its glycosidic bonding and the molecular chain binding substances [49].

#### 3.3.2. In Vitro Probiotic Activity

**Figure 5:**
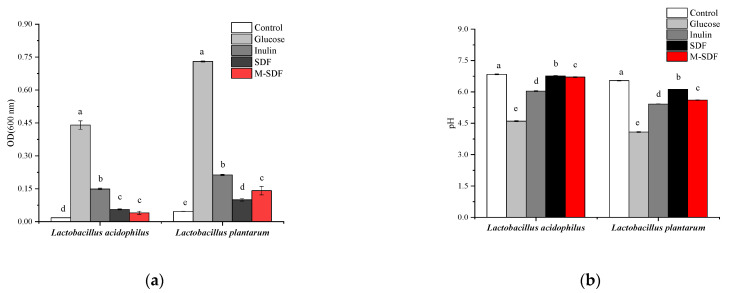
(**a**) OD value of MRS medium; (**b**) pH value of MRS medium. Different letters (a, b, c, d, e) in the same row indicate significantly different means at *p* < 0.05 (Duncan’s test).

In this study, we evaluated the ability of *Lactobacillus acidophilus* and *Lactobacillus plantarum* to utilize SDF and M-SDF as their carbon sources in glucose-free MRS base medium in comparison with inulin or glucose as sole carbon source. As showed in Figure 5, the growth of *Lactobacillus acidophilus* and *Lactobacillus plantarum* in the MRS medium supplemented with glucose was significantly higher than other media after 24 h of fermentation. The depletion of carbon sources promoted the growth of probiotics and correspondingly lowered the pH of the medium. Our results were similar to that of Chen et al. [50]. SDF and M-SDF significantly improved the growth of two bacterial species as compared to the sugar-free MRS medium, but its probiotic effect was weaker than that of inulin. In addition, M-SDF promoted the growth of *Lactobacillus plantarum* better than SDF at the concentration of 2.0%, which may have been due to the complex microstructure and the different monosaccharide ratios that affect accessibility and fermentability of dietary fiber. In living organisms, most of glucose is expected to be absorbed into the blood, rather than stimulating bacterial growth in the large intestine [51]. Related studies have shown that dietary fiber can resist the hydrolysis by digestive enzymes in the upper gut and undergo fermentation in the lower gut by the gut microbiota, stimulating the growth and activity of beneficial bacterial populations [52,53]. Therefore, it could be speculated that SDF and M-SDF are good substrates for promoting probiotic growth.

## 4. Conclusions

This study reports the first data in the literature for the dietary fiber samples obtained from litchi pomace and modified by ultrasonic enzymatic treatment, contributing to better understand the structural and functional properties of litchi pomace dietary fibers. The results indicated that a total of eight monosaccharides were detected in the four dietary fiber samples, with arabinose being the most abundant. All dietary fiber samples had the typical polysaccharide functional groups and different morphology. Additionally, SDF exhibited superior SC and WS, while IDF exhibited greater WHC. Compared to other fibers, the polysaccharide constituents of litchi pomace dietary fibers were strongly hydrophilic, and those materials might be well suited for formulated foods to reduce calories, avoid syneresis, prolong stability, and modify the texture. Moreover, soluble dietary fiber content of litchi pomace was successfully increased with enhanced arabinose content and apparent viscosity after ultrasonic enzymatic modification. Both SDF and M-SDF exhibited superior radical scavenging ability and potential as a source of prebiotics. Considering their ideal functional properties in vitro, we suggest soluble dietary fiber from litchi pomace could be exploited as an ingredient in functional foods. The functional activity in vivo of litchi pomace dietary fiber and its physicochemical and nutritional interactions with other ingredients in the food system are worthy of further study.

## Figures and Tables

**Table 1:** Monosaccharide compositions of SDF, M-SDF, IDF, and M-IDF.

mg/g	SDF	M-SDF	IDF	M-IDF
Mannose	37.49 ± 0.16 ^a^	27.22 ± 0.69 ^b^	26.63 ± 1.27 ^b^	25.44 ± 1.29 ^b^
Ribose	0.74 ± 0.19 ^a^	0.16 ± 0.01 ^b^	0.41 ± 0.04 ^b^	0.25 ± 0.02 ^b^
Rhamnose	17.81 ± 0.13 ^a^	18.63 ± 0.65 ^a^	8.43 ± 0.49 ^b^	5.86 ± 0.18 ^c^
Galacturonic acid	111.87 ± 4.54 ^b^	133.27 ± 6.46 ^a^	25.38 ± 1.27 ^c^	11.27 ± 0.48 ^d^
Glucose	44.40 ± 1.87 ^c^	79.17 ± 0.55 ^a^	50.14 ± 0.98 ^b^	49.09 ± 1.88 ^b^
Galactose	41.26 ± 0.14 ^a^	30.73 ± 1.09 ^b^	20.55 ± 0.27 ^c^	15.75 ± 0.15 ^d^
Xylose	3.21 ± 0.06 ^d^	7.03 ± 0.10 ^c^	21.90 ± 0.38 ^a^	12.32 ± 0.86 ^b^
Arabinose	187.02 ± 2.17 ^b^	233.11 ± 1.84 ^a^	102.00 ± 0.47 ^c^	83.14 ± 0.39 ^d^

Different letters (a, b, c, d) in the same row indicate significantly different means at *p* < 0.05 (Duncan’s test).

**Table 2:** Hydration properties of SDF, M-SDF, IDF, and M-IDF.

mg/g	SDF	M-SDF	IDF	M-IDF
WHC (g/g)	6.75 ± 0.09 ^c^	6.9 ± 0.13 ^c^	9.69 ± 0.2 ^a^	9.07 ± 0.09 ^b^
SC (mL/g)	8.63 ± 0.18 ^a^	7.75 ± 0.35 ^b^	6.65 ± 0.28 ^c^	7.18 ± 0.25 ^b,c^
WS (%)	83.1 ± 1.76 ^a^	78.34 ± 0.2 ^b^	10.22 ± 0.17 ^c^	9.29 ± 0.32 ^c^

Different letters (a, b, c) in the same row indicate significantly different means at *p* < 0.05 (Duncan’s test).

## Data Availability

All data from this study have been reported in the manuscript.
